# Identifying and treating older patients with malnutrition in primary care: the MUST screening tool

**DOI:** 10.3399/bjgp18X697853

**Published:** 2018-07

**Authors:** Jane Murphy, Alice Mayor, Emer Forde

**Affiliations:** Faculty of Health and Social Sciences; centre for general practice, Bournemouth University, Bournemouth.; Poole Town Surgery, Poole.; Centre for general practice, Bournemouth University, Bournemouth.

## INTRODUCTION

Malnutrition means ‘poor nutrition’ when there is a deficiency of energy and nutrients in the diet that causes a measurable clinical outcome or impact on body composition or function.^1^ As such, malnutrition can refer to people who are overweight but the term is more often used to apply to people who are underweight. Malnutrition (as undernutrition) affects an estimated 3 million people in the UK (those either malnourished or at risk), the majority of whom are living in the community (93%).^2^ It can be a cause and consequence of illness, and is a common clinical and public health problem that has largely been ignored in primary care.^3^^,^^4^ This may be because the reverse problems of overeating, and obesity, have been the focus of attention in regard to weight management and nutritional status.

Malnutrition can have a profound detrimental impact on disease risk, progression, and prognosis, as well as increasing complications after injury and delaying recovery from illness. It results in greater healthcare needs in the community, including more frequent access to GP services and increased needs at home. Recent estimates suggest that the health and social care cost of malnutrition in both adults and children in England alone exceeds £19 billion, and half of this expenditure is due to people >65 years.^3^ Overall, the cost of treating a malnourished patient is over three times more than treating a non-malnourished patient. It is only set to increase with an ageing population and the rising cost of health and social care services.

## SCREEN AND TREAT

Older patients as a group are particularly at risk, and it is a common misbelief that weight loss is an inevitable part of the ageing process. Malnutrition exacerbates the age-related decline in muscle mass and strength, leading to sarcopenia, which can contribute to frailty.^5^ Thus early identification and treatment can significantly reduce the clinical risks associated with malnutrition, including vulnerability to illness, clinical complications, and mortality. As most malnutrition originates in the community, early identification should be undertaken in the primary care setting.

National Institute for Health and Care Excellence (NICE) guidelines recommend that GPs screen patients on registration and when there is cause for any clinical concern.^6^^,^^7^ Patients should also be screened opportunistically, for example, at routine health checks and when attending for flu jabs. Patients in care homes should also be screened. NICE guidelines specifically recommend that patients should be weighed, their body mass index (BMI) calculated, and screening should include a validated tool such as the Malnutrition Universal Screening Tool (MUST).^2^

The MUST was developed by the multidisciplinary Malnutrition Advisory Group of the British Association for Parenteral and Enteral Nutrition (BAPEN).^2^^,^^8^ Three independent criteria are used:

- current weight status using body mass index;
- unintentional weight loss — there is evidence that a weight loss of 5–10% can produce physiologically relevant changes in body function;^2^ and
- acute disease effect producing or likely to produce no nutritional intake for >5 days.

Each parameter is scored on a scale of 0–2. Based on the score, the patient is defined as belonging to a low (0), intermediate (1), or high (≥2) risk of malnutrition group. Those identified at medium or high risk should have a nutrition management care plan devised using the malnutrition pathway.^9^ This helps define which patients need specialist dietary advice, assistance with meals, food first strategies, or prescriptions for oral nutritional supplements (ONS). Local guidelines may vary but GPs should be able to request protocols from their community dieticians. As an example, this is the referral pathway in Dorset.^10^ Those identified at medium or high risk should be given clear goals in their care plans including a date to review their nutritional status.

## WHAT’S BEEN DONE IN PRIMARY CARE?

Despite these recommendations, it would appear that malnutrition has attracted little attention in primary care, and screening using validated tools is not routinely included in care planning for over-75s. The reasons for this could include lack of knowledge and training within primary care; a focus on overeating and obesity as the major health need for the population; lack of leadership or ownership from GPs who perhaps regard this as a role for practice nurses or dieticians; lack of resources; funding pressures; and an over-burdened clinical agenda.^3^

Two authors undertook a retrospective review in their own practice, which is a small GP surgery in Dorset with a relatively high population of older people, with 539/3797 (14.2%) registered patients >75 years. It is an accredited ‘Dementia Friendly’ surgery, and is a teaching practice with high Quality and Outcomes Framework ratings and good patient satisfaction ratings. Of the 539 patients, there were 411 (76%) who had a BMI recorded in the last 2 years, and 12 (3%) of these patients were underweight (BMI under 18.5 kg/m^2^). Of the 12 underweight patients, only one (8%) had a MUST score undertaken and four (33%) had documentation of receiving dietary advice; three (25%) had been referred to a dietician and four (33%) were prescribed ONS. Within this group, there were high rates of primary care consultations, with an average of 12 nurse contacts/year, due largely to high rates of pressure sores and ulcers in these patients. These data are consistent with other reports that show patients are not being adequately screened for malnutrition using validated tools (such as MUST), and clinical management could be better.

## CONCLUSION

Systematic screening and appropriate management of malnutrition in primary care are significant clinical issues that have hitherto largely been ignored in primary care. Measuring BMI is not adequate and it is important that validated tools for malnutrition, such as MUST, are utilised. This will improve nutritional care, help identify those at risk, and determine who to treat with supplements and/or professional dietary advice. There is a need to raise awareness of malnutrition in the community, the impact on patient outcomes, and the economic cost to the NHS. GPs need access to appropriate training, primary care teams need to decide who takes the lead on managing patients’ nutritional needs (GPs, nurses, or dieticians), and commissioners need to consider incentives,^11^ local protocols, and resources. We suggest that, as new models of care are being developed, there is an opportunity to integrate community dieticians more fully within the primary healthcare team.

To date, there have been a number of initiatives to address the problem of malnutrition such as the work undertaken by the Malnutrition Task Force (http://www.malnutritiontaskforce.org.uk/) and BAPEN (http://www.bapen.org.uk/).

The Nutrition in Older People Programme delivered by the Wessex Academic Health Science Network has been evaluating new integrated approaches and strategies for the identification and treatment of malnutrition in the community and has developed new tools and resources as part of its Older People’s Essential Nutrition (OPEN) toolkit. Materials are free to download.^12^
